# C1QBP Mediates Breast Cancer Cell Proliferation and Growth via Multiple Potential Signalling Pathways

**DOI:** 10.3390/ijms24021343

**Published:** 2023-01-10

**Authors:** Olivia J. Scully, Sukanya Shyamasundar, Ken Matsumoto, S. Thameem Dheen, George W. Yip, Boon Huat Bay

**Affiliations:** 1 Department of Anatomy, Yong Loo Lin School of Medicine, National University of Singapore, Singapore 117594, Singapore; 2 Chemical Genomics Research Group, RIKEN Center for Sustainable Resource, 2-1 Hirosawa Wako-shi, Saitama 351-0198, Japan

**Keywords:** C1QBP, carcinogenesis, gene expression, pathway enrichment, targeted therapy

## Abstract

Breast carcinoma is the most prevalent cancer in women globally, with complex genetic and molecular mechanisms that underlie its development and progression. Several challenges such as metastasis and drug resistance limit the prognosis of breast cancer, and hence a constant search for better treatment regimes, including novel molecular therapeutic targets is necessary. Complement component 1, q subcomponent binding protein (C1QBP), a promising molecular target, has been implicated in breast carcinogenesis. In this study, the role of C1QBP in breast cancer progression, in particular cancer cell growth, was determined in triple negative MDA-MB-231 breast cancer cells. Depletion of C1QBP decreased cell proliferation, whereas the opposite effect was observed when C1QBP was overexpressed in MDA-MB-231 cells. Furthermore, gene expression profiling and pathway analysis in C1QBP depleted cells revealed that C1QBP regulates several signaling pathways crucial for cell growth and survival. Taken together, these findings provide a deeper comprehension of the role of C1QBP in triple negative breast cancer, and could possibly pave the way for future advancement of C1QBP-targeted breast cancer therapy.

## 1. Introduction

Breast cancer is the most common cancer in women globally, constituting a quarter of newly diagnosed cancers and the main cause of mortality in women afflicted with the disease in 2020 [1]. The global breast cancer burden is increasing at an alarming rate and it is predicted that by 2040, 3 million new breast cancer cases per year are expected with an associated 1 million deaths that could occur up from the incidence of 2.2 million new cases and 680,000 deaths in 2020 [2].

Breast carcinoma, a complex disease, has been classified into different subtypes depending on the expression of molecular markers and pathological signatures. Triple negative breast cancers (TNBCs), a basal-like breast cancer constitute ~1/5th of the breast carcinomas [3], and do not express receptors for estrogen/ progesterone hormones or the human epidermal growth factor receptor 2 [4,5]. Six molecular TNBC subtypes have been identified recapitulating the heterogeneity of this group of breast cancers [6]. TNBCs demonstrate highly aggressive and invasive characteristics, with the brain, lung and liver as frequent sites of metastasis [7]. The current standard therapeutic option available for patients with TNBC is chemotherapy as they lack specific receptors for effective targeted therapy. Despite conventional therapy, the overall survival of TNBC patients is shorter as a result of metastasis and treatment failure due to chemoresistance [8]. Recently, several novel agents have been developed to treat breast cancer [9], but there is still a pressing need for efficacious therapeutic strategies to increase the survival of these patients. The advent of precision oncology has led to the discovery of dysfunctional signaling pathways and anomalous protein expression in patients with TNBC, which have shown promise as novel therapeutic targets [10].

Aberrant proliferation that occurs due to the dysregulation of cell cycle proteins, is a hallmark of cancer cells including that of the breast. Thus, assessment of cell proliferation is crucial for prognostication and treatment planning for breast cancer patients [11]. Complement component 1, q subcomponent binding protein (C1QBP, also known as p32 or hyaluronan binding protein 1 or globular C1q binding protein), which is predominantly localized in the mitochondria, has been associated with several cancers including the ovary, endometrium, breast, prostate and pancreas (reviewed in [12]). High expression of C1QBP has been observed to be a potential breast cancer prognostic marker for regional spread to axillary lymph nodes, distant metastasis, tumor recurrence and poor survival [13,14,15]. In addition, this multifunctional protein has been identified as a potential therapeutic target, as several studies have demonstrated that knockdown of C1QBP inhibit proliferation, migration and metastasis of breast cancer cells [16,17,18,19]. Furthermore, preliminary studies have shown that C1QBP targeted therapy, such as using anti-C1QBP antibodies in breast cancer [20], and C1QBP inhibitor in glioma [21] have a potential therapeutic value. Therefore, a thorough investigation of the molecular pathways that underlie C1QBP-mediated tumor progression is crucial for providing biological and mechanistic insights, that would be valuable for developing effective treatment strategies.

In this study, we examined the functional role of C1QBP in promoting the cellular proliferative process and identified potential signaling pathways that could mediate C1QBP-associated tumor growth and progression in breast cancer.

## 2. Results

### 2.1. C1QBP Is Mainly Localized to Mitochondria in the Cytoplasm and Also Present in Cell Nuclei

**Figure 1:**
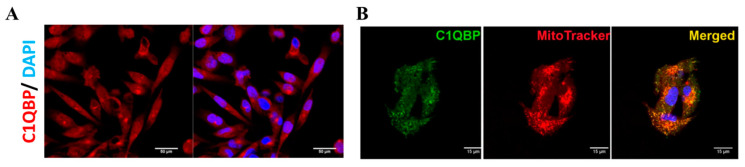
(**A**) Confocal microscopy showing mainly cytoplasmic C1QBP staining and also nuclear staining in a fraction of MDA-MB-231 cells. Scale bar: 50 μm. (**B**) Merged panel (yellow) depicts the co-localization of C1QBP (green) with MitoTracker^®^ Red CMXRos (red). Scale bar: 15 μm.

MDA-MB-231 breast cancer cells were selected for experimentation since it is known to have a high expression of C1QBP [13]. Immunostaining revealed intracellular localization of C1QBP in both the cytoplasm (speckled appearance) and nuclei of the MDA-MB-231 cells (Figure 1A). Mitochondrial localization was verified by C1QBP and MitoTracker^®^ Red CMXRos immunoflurorescence staining. Indeed, C1QBP (green fluorescence) was noted to be predominantly co-localized with Mitotracker red in the MDA-MB-231 cells as shown in Figure 1B.

### 2.2. Silencing of C1QBP Decreases Cell Growth

**Figure 2:**
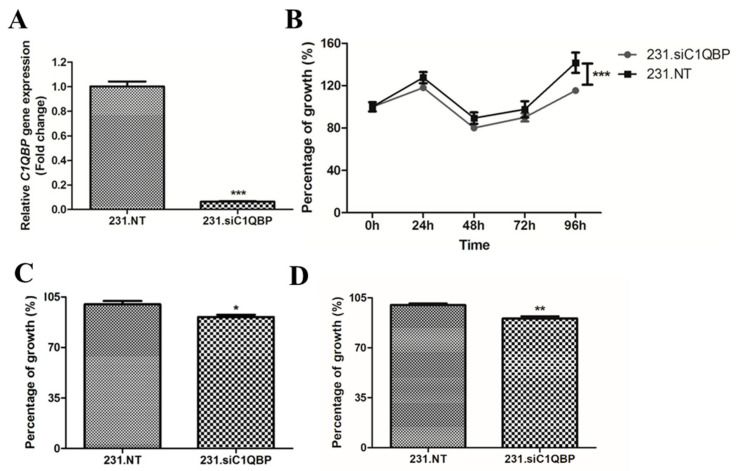
(**A**) *C1QBP* mRNA expression in knockdown and control MDA-MB-231 cells. *C1QBP* gene expression was decreased by 94% in cells 48 h post-transfected with C1QBP siRNA. *** *p* < 0.001. (**B**) Downregulation of the C1QBP protein had a profound negative effect on cell growth as analysed by two-way ANOVA (*** *p* < 0.001). MTS assay showed that knockdown of *C1QBP* decreased cell proliferation at (**C**) 72 h and (**D)** 96 h after transfection. * *p* < 0.05, ** *p* < 0.01.

Following siRNA mediated knockdown, *C1QBP* mRNA in MDA-MB-231 cells (231.siC1QBP) was found to be reduced by >90% at 48 h following transfection when compared with control (231.NT) cells (Figure 2A). Next, a growth curve was generated in *C1QBP* silenced MDA-MB-231 cells over a period of 96 h (Figure 2B). 231.siC1QBP cells showed significantly lower cell growth in comparison with control cells. In addition, a 3-(4,5-dimethylthiazol-2-yl)-5-(3-carboxymethoxyphenyl)-2-(4-sulfophenyl)-2H-tetrazolium (MTS) assay was performed to measure cell proliferation after silencing of the *C1QBP* gene. Cells were first synchronised by fasting for 24 h and the MTS assay performed at 72 h and 96 h post transfection. Our results showed that *C1QBP*-silenced cells had a significant reduction in cell proliferation at both 72 h and 96 h post transfection, thus verifying the findings of the growth curve analysis (Figure 2C,D).

### 2.3. Overexpression of C1QBP Enhances Cell Growth

**Figure 3:**
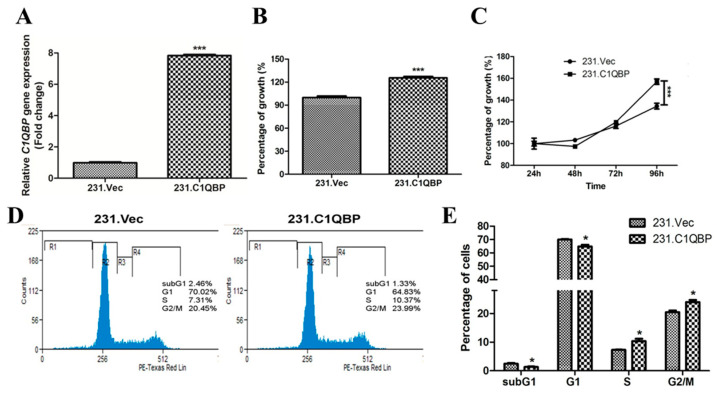
(**A**) C1QBP overexpression in MDA-MB-231 cells. qRT-PCR showed significant up-regulation in *C1QBP* mRNA expression in C1QBP-overexpressing cells. *** *p* < 0.001 (**B**,**C**) Stable overexpressing C1QBP cells enhanced cell growth. (**B**) MTS assay showed substantial increase in cell proliferation in C1QBP overexpressing cells. *** *p* < 0.001. (**C**) Growth curve analysis using AlamarBlue assay on overexpressing C1QBP cells revealed an increased growth rate as analysed by two-way ANOVA, *** *p* < 0.001. (**D**,**E**) Overexpression of the *C1QBP* gene increased the percentage of cells in S and G2/M phases. (**D**) Representative images of the cell cycle profiles of 231.Vec cells and 231.C1QBP cells, respectively, obtained by flow cytometry. (**E**) Cell cycle phases expressed in percentages. Values presented as mean ± SEM. * *p* < 0.05.

For stable overexpression, pCI-neo-C1QBP plasmid or empty pCI-neo plasmid was transfected into the cells. Stable overexpressing cells had eight-folds higher *C1QBP* gene expression than cells transfected with empty plasmids (Figure 3A). An MTS assay revealed that MDA-MB-231 cells had increased cell growth at 72 h post seeding following C1QBP overexpression (Figure 3B). An AlamarBlue assay performed over 96 h also showed a remarkable increase in cell growth following C1QBP overexpression (Figure 3C).

Cell cycle analysis was then performed at 72 h after seeding (Figure 3D). The results revealed that C1QBP overexpression induced a considerable reduction of cells in the sub-G1 phase (2.46% vs. 1.33% for 231.Vec cells compared with 231.C1QBP cells) and the G1 phase (64.83% vs. 70.02% for 231.C1QBP cells when compared to control vector-transfected cells) (Figure 3E). Concomitantly, C1QBP overexpression increased the percentage of cells in the S phase (7.31% vs. 10.37%, for 231.Vec cells compared with 231.C1QBP cells) and G2/M phase (20.45% vs. 23.99%, for 231.Vec compared with 231.C1QBP). Taken together, the results signify that the C1QBP protein augmented cell cycle progression.

### 2.4. Genome-Wide Analysis of C1QBP-Silenced MDA-MB-231 Cells Revealed Potential Signaling Pathways Regulated by C1QBP in Breast Cancer

**Figure 4:**
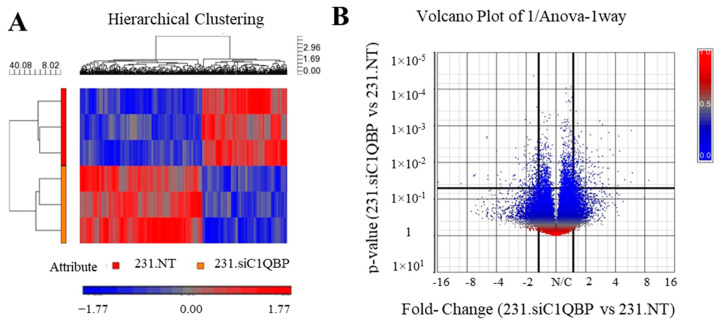
(**A**) Hierarchical clustering concurrent with (**B**) volcano plot of the genes obtained from the Affymetrix gene chip. Thick vertical lines and horizontal lines in the volcano plot represent the chosen cut off of fold change of 1.5 and *p* < 0.05.

Gene expression profiling was carried out to ascertain the mechanistic pathways underlying the functional changes following *C1QBP* silencing in MDA-MB-231 cells. Hierarchical clustering showed that 231.siC1QBP replicates had distinct expression from the replicates of control 231.NT cells (Figure 4A). A volcano plot was generated to depict changes in gene expression following *C1QBP* knockdown (Figure 4B). A significant number of genes (186 genes) were observed to be differentially expressed, with 77 genes significantly up-regulated in expression whereas 109 genes showed decreased expression. A list of dysregulated genes is annotated in Supplementary Table S1. For validation, expression of 15 genes were also determined by real-time PCR, and a majority of the genes analysed were observed to be consistent with the microarray data (Supplementary Figure S1).

**Figure 5:**
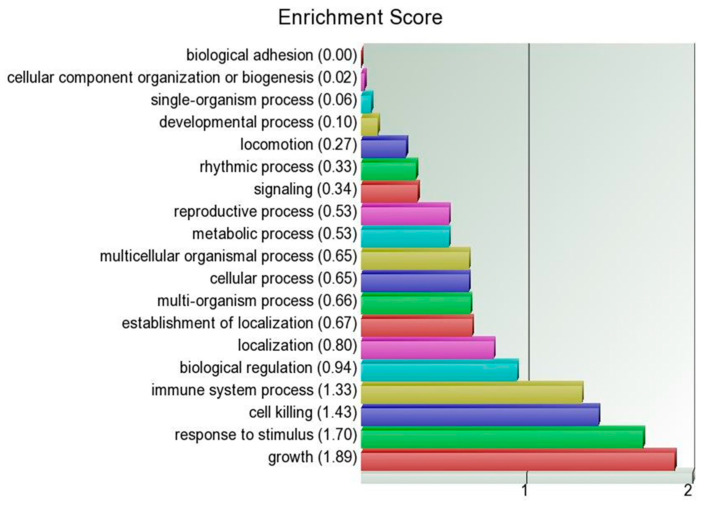
Enrichment scores for biological functions of differentially expressed genes in C1QBP silenced breast cancer cells derived from the Partek Genomics Suite 6.6 software (Partek Inc., St. Louis, MI, USA).

The biological functions of the differentially expressed genes are shown in Figure 5.

Interestingly, cell growth function received the highest enrichment score suggesting that *C1QBP* silencing affected several genes involved in cell growth. In addition, the differentially expressed genes based on their functions were categorized by the Database for Annotation, Visualization, and Integrated Discovery (DAVID) software (Table 1 and Supplementary Table S2). In addition to cell cycle and cell death (Table 1), genes were categorized into functional processes such as gene transcription, enzymatic activity, stress response and others (Supplementary Table S2).

Moreover, pathway analysis identified genes involved in the degradation of fatty acids and branched chain amino acids (BCAAs), as well as hypoxia-inducible factor 1 (HIF-1) signaling pathway among others (Table 2). Notably, expression of both *Acetyl-CoA Acyltransferase 1* (*ACAA1*) and *Glutaryl-CoA Dehydrogenase* (*GCDH*) mRNAs involved in the degradation of fatty acids and BCAAs were decreased after knockdown of *C1QBP* (Full list of pathways shown in Supplementary Table S3).

## 3. Discussion

Metastatic breast cancer has a poor prognosis with a median survival of 1.5–5 years despite therapy, whereas the TNBC subtype has the worst prognosis compared to other subtypes with an overall survival of 8–13 months [22], and progression free survival of 3–4 months [23]. Therefore, developing reliable prognostic biomarkers and novel targeted therapy is still much needed in patients with TNBC.

A hallmark of cancer is cell proliferation, which drives tumor growth and progression [24]. In normal tissues and organs, the balance between cell death and organized cell proliferation regulates cell growth [25]. However, in cancer cells, aberrant genetic and epigenetic alterations in the expression of oncogenes and tumor suppressor genes is beneficial for the growth of cancer cells [26]. Furthermore, cell proliferation is a crucial indicator for tumor prognosis in standard breast cancer therapies. In particular, cell proliferation is used to evaluate treatment in the luminal receptor-positive subtype of breast cancer in males [27]. In this present study, silencing of *C1QBP* in triple negative MDA-MB-231 breast cancer cells was concomitant with reduced cell proliferation and a diminished rate of cell growth. Our results also corroborate with previous reports, wherein downregulation of C1QBP was observed to decrease cell proliferation in breast and prostate cancer cells [18,28]. Stable knockdown of the *C1QBP* gene has also been previously shown to inhibit cell proliferation in MDA-MB-231 breast cancer cells [18]. On the other hand, as demonstrated in our present study, overexpression of the C1QBP protein elicited the opposite effect by enhancing cell proliferation in the breast cancer cells. Similarly, overexpression of C1QBP in liver carcinoma cells has likewise been reported to increase cell proliferation [29].

Furthermore, overexpression of C1QBP in this present study led to G1 to S phase progression in MDA-MB-231 cells, signifying that cells are progressing to a proliferative stage. In concurrence, *C1QBP* silencing was reported to induce G1 to S phase arrest in prostate cancer cells [28]. An uncontrolled cell cycle that is observed in cancer cells is e caused by dysregulated growth signals, and as a consequence may affect other cellular characteristics such as cell survival [24]. As the G1 phase is a crucial phase during the cell cycle [30], unrestricted G1 to S phase transition in cancer cells will lead to accelerated cell maturation and differentiation [31].

Tumor progression is due to uncontrolled cell growth, and a consequence of dysregulated genetic and epigenetic mechanisms. Various signalling pathways that regulate cell growth, proliferation, migration and survival underlie aberrant tumor growth. Furthermore, dysregulated signalling transduction pathways also impact wider signalling networks, leading to an altered tumor microenvironment, angiogenesis and inflammation and may thus promote tumor progression [32]. Therefore, we carried out gene expression profiling to decipher the role of C1QBP in metastatic breast cancer and identify potential cancer signalling transduction pathways. The enrichment scores revealed that several genes involved in cell growth and survival were differentially expressed following *C1QBP* silencing in MDA-MB-231 cells. Consistent with this line of investigation, we have previously shown that C1QBP is a proliferative marker in breast cancer tissue samples, and depletion of C1QBP protein in other breast cancer sub-types such as progesterone receptor positive T47D cells decreased cell proliferation and growth [13].

Pathway enrichment of C1QBP depleted MDA-MB-231 cells revealed that fatty acid degradation (and synthesis), degradation of BCAAs, adipocytokine signalling and mRNA surveillance are the top pathways among many others. Decreases in *ACAA1* (also associated with peroxisome proliferator-activated receptors signalling) and *GCDH* mRNAs in C1QBP-depleted cells may implicate the impairment of fatty acid beta-oxidation in peroxisomes and BCAA degradation in mitochondria, both of which are known to provide substrates for the citric acid cycle. Depletion of C1QBP has been shown to induce mitochondrial dysfunction and the morphological changes in mitochondria [17,33]. It is well-established that cancer cells undergo metabolic reprogramming in order to increase the synthesis of carbohydrates and amino acids to aid cell proliferation [34]. Alterations in lipid metabolism have also been shown to drive migration, invasion and angiogenesis of several cancers including the breast [35,36]. In addition, C1QBP has been linked with lipid metabolism and its depletion reduces fat synthesis and utilization [33,37,38]. Very recently, C1QBP has been associated with fatty acid degradation, where silencing of *C1QBP* has been shown to reduce lipid droplets and triglycerides in prostate cancer cells [39]. BCAAs are essential amino acids (such as isoleucine, valine and lysine) that are derived from the tumor microenvironment or degradation of proteins, and recent reports suggest that BCAAs drive tumor development and progression by activating the mTORC1 signalling pathway [40]. Furthermore, in pancreatic cancer, BCAAs have been reported to control cell proliferation, where reduction of BCAAs decreased proliferation and fatty acid expression [41]. Similarly, some BCAA metabolizing enzymes have been identified as prognostic marker in several cancers including the breast (reviewed in [42]). It is also fascinating that apolipoprotein A-I, a high density lipoprotein component, which has antitumor properties and is known to be involved in cholesterol transport and tissue efflux, was shown to be an interacting partner of C1QBP [43].

There were also other pathways that were identified. For instance, HIF-1α has been associated with growth, metastasis and treatment resistance in breast cancer by activating the PI3K/Akt/mTOR pathway [44]. C1QBP has also been implicated in Akt/mTOR signaling, which affects the migration capability of colon cancer cells [45]. TNBCs are reported to have increased expression of HIF-1α [46] and recently, Wu et al. showed that HIF-1α upregulates C1QBP in TNBCs [47]. The same investigators observed that C1QBP modulate metastatic processes in Hs578T and MDA-MB-468 breast cancer cells and in breast cancer in vivo via NF-kappa B signaling [47], a pathway which was also picked up in the current study (Supplementary Table S3). Forkhead box O regulated genes, such as *GABA type A receptor associated protein like 1* (*Gabarapl1*), which is important for autophagy, cell proliferation and survival, and has been identified as a prognostic marker for breast cancer where elevated expression of *Gabarapl1* mRNA levels were associated with lower metastasis risk in lymph node positive patients [48]. The pro-inflammatory interleukin (IL-23A) signalling cascade involves the activation of the Janus kinase-signal transducer and an activator of transcription (JAK-STAT), which are essential for cell proliferation and survival [49]. IL-23 has also been reported as a prognostic breast cancer marker [50] and a potential therapeutic agent. Interestingly, although C1QBP has been reported to modulate cell adhesion and migration in renal cell cancer by regulating the cell adhesion molecule L1CAM via the Wnt/β-catenin pathway [51], and promote metastasis in pancreatic cancer via the insulin-like growth factor (IGF) signalling pathway [52], both pathways were not identified from the bioinformatics analysis in the present study. The findings suggest the possibility that cellular signalling pathways mediated by C1QBP may also differ depending on the organ of origin of the cancer.

## 4. Materials and Methods

### 4.1. Cell Culture

MDA-MB-231 breast cancer cells were obtained from American Tissue Culture Collection (ATCC, Manassas, VA, USA) and cultured according to routine protocol. Cells were passaged when 80% confluent using 1X Trypsin-EDTA (Invitrogen, Carlsbad, CA, USA).

### 4.2. Gene Silencing and Overexpression

For gene silencing, cells were seeded in 6-well plates (2.5 × 10^5^/well) for 24 h. In total, 20 nM of ON-TARGETplus SMARTpool siRNA targeting C1QBP or non-targeting siRNA pool was transfected into the cells using DharmaFECT2 obtained from GE Dharmacon (Lafayette, CO, USA). The ID and target sequences for the 4 C1QBP siRNAs present in the pool are as follows: J-011225-13 (GCGAAAUUAGUGCGGAAAG), J-011225-14 (CGCAAGGGCAGAAGGUUGA), J-011225-15 (UUUCGUGGUUGAAGUUAUA), and J-011225-16 (GAAGUUAGCUUUCAGUCCA). After 24 h, the culture media was replenished and cells were harvested.

pCI-neo-C1QBP was used for overexpression of C1QBP. pCI-neo-C1QBP was generated by insertion of full-length human C1QBP cDNA (containing a C-terminal c-myc tag), into a pCI-neo plasmid. A complex of 1 µg of pCI-neo-C1QBP or pCI-neo plasmids with Lipofectamine 2000 (Thermos Fisher Scientific, Waltham, MA, USA) was used for transfection. Neomycin-resistant colonies were selected using 400 µg/ml. Geniticin^®^ (Gibco, Grand Island, NY, USA).

### 4.3. Immunocytochemistry

Cells were grown on coverslips or chambered coverglass until reaching ~80% confluence. Subsequently, 4% paraformaldehyde was added to fix the cells. Following fixation, the cells were incubated in 0.1% Triton X-100 for permeabilisation. Non-specific binding was reduced by incubating the cells with 1% BSA, following which rabbit anti-C1QBP antibodies [53] were added, and incubation was carried out overnight at 4 °C. The next day, cells incubated with secondary anti-rabbit-FITC or CY3 conjugated antibody (Sigma-Aldrich, St. Louis, MO, USA) at room temperature for 1 h. Finally, the cells were counterstained and mounted using Vectashield^®^ fluorescence mounting medium which contained 4′,6-diamidino-2-phenylindole (DAPI). For mitochondrial staining, 250 nM MitoTracker^®^ Red CMXRos (Invitrogen) was added to the culture medium, and incubated for 30 min at 37 °C before fixation and immunostaining. Finally, slides were viewed on the Olympus Fluoview FV1000 Laser Scanning Confocal Microscope (Tokyo, Japan).

### 4.4. RNA Isolation and PCR

Total RNA from C1QBP silenced or overexpressed cells were harvested using the RNeasy Minikit (Qiagen, Hilden, Germany), following the manufacture’s protocol. Quality of RNA and concentration of RNA were measured on a Thermo Fisher Nanodrop Spectrophotometer (Waltham, MA, USA). A total of 1–2 µg of total RNA was converted to cDNA and the expression of *C1QBP* was analysed by real-time PCR with *GAPDH* as the housekeeping gene. The 2^−ΔΔCt^ method was used to determine the relative expression of the *C1QBP* gene. The primer sequences for *C1QBP* and *GAPDH* have been detailed previously [13].

### 4.5. Cell Proliferation Assays

MTS assay and AlamarBlue assay were performed to measure cell proliferation or cell growth following silencing/overexpression of C1QBP as detailed previously [13,54]. After 48 h post-transfection, cells were kept in serum free medium for 24 h and then re-supplemented with FBS, for either 24 h (silencing experiment) or 48 h (overexpression experiment) before performing the proliferation assay.

### 4.6. Flow Cytometry Analysis

C1QBP overexpressed cells and control cells were harvested by centrifugation. Fixation of cells were carried out using ice-cold 70% ethanol at 4 °C overnight. Subsequently, cells were labelled using a mixture of Propidium iodide, RNase A and 0.1% Triton-X 100, before analysis in a flow cytometer (Beckman Coulter, Inc., Indianapolis, IN, USA). Data was processed on the Summit software V4.3 (Beckman Coulter).

### 4.7. Gene Expression Microarray and Pathway Analysis

48 h post-transfection of C1QBP or NT siRNA and total RNA was collected from the cells. The knockdown efficiency was ascertained by qRT-PCR and then the samples were further processed by Origen Labs (Singapore). The concentration of RNA was determined using the BioSPEC-Mini spectrophotometer (Shimadzu Corporation, Kyoto, Japan), whereas the RNA integrity number was analysed on the Bioanalyzer (Agilent technologies, Santa Clara, CA, USA). The samples were processed based on the Affymetrix and NuGEN recommended protocols or Origen Laboratory’s Standard Operating Procedure. Briefly, 100 ng of total RNA was used to produce double- stranded cDNA by reverse transcription. Single stranded anti-sense cDNA was generated by Single Primer Isothermal Amplification (SPIA), and biotin-labelled sense cDNA generated by post-SPIA modification. Following this, the cDNA was hybridized to the Affymetrix Human Gene 2.0 ST array (Affymetrix, Santa Clara, CA, USA) for 18 h at 45 °C. Subsequently, washing and staining was done following the FS450_0007 fluidics protocol, and the arrays were scanned using an Affymetrix 3000 7G scanner. Expression Console (EC) 1.1 software was used to determine the quality control.

Genome-wide gene expression between siC1QBP and NT siRNA transfected MDA-MB-231 cells were compared. The CEL files were analysed in the GeneSpring 11.5 software (Silicon Genetics, Redwood City, CA, USA) and the Partek Genomics Suite (Partek Inc., St. Louis, MI, USA). Differentially expressed genes were determined by using a cut-off fold change of ±1.5 and *p* value < 0.05. Pathway enrichment was done using the DAVID software v 6.7 alongside the Partek Suite.

### 4.8. Statistical Analysis

Results were analysed in GraphPad Prism 5 (San Diego, CA, USA) using two-tailed student *t*-test, one-way ANOVA and two way ANOVA. Data are represented as mean ± SEM and *p* < 0.05 was considered as statistically significant. All experiments were carried out with at least triplicates and repeated at least twice.

## 5. Conclusions

Overall, our results together with others provide further evidence that C1QBP has a significant role in maintaining metabolic activities, and that its depletion leads to alterations in cellular metabolism and reduced cell proliferation. Thus far, several targeted agents such as inhibitors for poly(ADP-ribose) polymerase, vascular endothelial growth factor receptors, epidermal growth factor receptors, ras/raf/mitogen activated protein kinase, phosphatidylinositol 3-kinase, IGF 1, histone deacetylase, and heat shock protein 90, are being explored as potential therapeutic targets for breast cancer, with many of them in clinical trials [9,55]. Given that C1QBP is associated with proliferation and implicated in lipid metabolism and BCAAs, future studies using clinical samples and cutting-edge omics technologies are necessary to pave the way for the identification of C1QBP-based theranostics for breast cancer, which may add to the therapeutic landscape of TNBC [56].

## Figures and Tables

**Table 1:** Categorization of genes associated with cell cycle and cell death using DAVID.

Function	Gene ID	Gene Name	Gene EntrezID
Cell cycle	UPF1	UPF1 regulator of nonsense transcripts homolog	5976
	BCL2	B-cell CLL/lymphoma 2	596
	MAP3K8	Mitogen-activated protein kinase kinase kinase 8	1326
	TREX2	Three prime repair exonuclease 2	11,219
	TOP3A	Topoisomerase (DNA) III alpha	7156
	PSMD9	Proteasome (prosome, macropain) 26S subunit, non-ATPase, 9	5715
Cell death	FGD1	FYVE, RhoGEF and PH domain containing 1	2245
	MICA	MHC class I polypeptide-related sequence A	4276
	NME3	NME/NM23 nucleoside diphosphate kinase 3	4832
	ZFYVE27	Zinc finger, FYVE domain containing 27	118,813
	BCL2	B-cell CLL/lymphoma 2	596
	GREM1	Gremlin 1, DAN family BMP antagonist	26,585
	PNPLA6	Patatin-like phospholipase domain containing 6	10,908
	GAL	Galanin/GMAP prepropeptide	51,083
	RASGRP1	RAS guanyl releasing protein 1 (calcium and DAG-regulated)	10,125

**Table 2:** Selected pathways for C1QBP-mediate cell proliferation and growth (with enrichment score > 1).

Pathway Name	Enrichment Score	Genes
Fatty acid degradation	3.51594	ACAA1, GCDH
Valine, leucine and isoleucine degradation	3.51594	ACAA1, PCCB
Adipocytokine signaling pathway	2.67827	POMC, SLC2A4
mRNA surveillance pathway	2.22979	PABPC1L, UPF1
HIF-1 signalling	1.97945	BCL2, HIF1A
Cell cycle	1.73155	CDC14B, MCM4
FoxO signaling pathway	1.62423	GABARAPL1, SLC2A4
Jak-STAT signaling pathway	1.37955	IL23A
mTOR signaling pathway	1.16873	HIF1A
PPAR signaling pathway	1.06625	ACAA1

## Data Availability

Data available from corresponding authors on reasonable request.

## Supplementary Materials

The following supporting information can be downloaded at: https://www.mdpi.com/article/10.3390/ijms24021343/s1.
