# Supplementary material for: C1QBP Mediates Breast Cancer Cell Proliferation and Growth via Multiple Potential Signalling Pathways
Source: Int J Mol Sci. 2023 Jan 10;24(2):1343. doi: 10.3390/ijms24021343 (PMC9864289; doi:10.3390/ijms24021343)
Supplement: Supplementary file 1 [file ijms-24-01343-s001.zip › Supplementary Tables S1, S2 and S3.pdf]

Table S1. List of differentially expressed genes

Transcript ID	Gene Symbol	Gene name	RefSeq	p-value (Attribute)	p-value (siybap1 vs. nt)	Ratio (siybap1 vs. nt)	Fold-Change (siybap1 vs. nt)	F(Attribute)	SS(Attribute)	SS(Error)	F(Error)
16761518	TAS2R31	Taste receptor, type 2, member 31	NM_176885	0.0130177	0.0130177	2.43929	2.43929	18.1771	2.48248	0.546288	1
16820947	HPR	Haptoglobin-related protein	NM_020995	0.0352038	0.0352038	2.26608	2.26608	9.79348	2.08931	0.853347	1
16924149	TPTE	Transmembrane phosphatase with tensin homology	ENST00000415664	0.0136987	0.0136987	2.16776	2.16776	17.6378	1.86888	0.423834	1
16801028	RNA5SP395	RNA, 5S ribosomal pseudogene 395	ENST00000516567	0.0499184	0.0499184	2.14439	2.14439	7.71751	1.81688	0.941693	1
16992195	MIR378E	MicroRNA 378e	NR_039609	0.0479144	0.0479144	2.13078	2.13078	7.94241	1.78666	0.899809	1
17051439	RNU7-73P	RNA, U7 small nuclear 73 pseudogene	ENST00000458743	0.0105224	0.0105224	2.06486	2.06486	20.5829	1.64131	0.318966	1
17008025	RAB44	RAB44, member RAS oncogene family	NM_001257357	0.00393404	0.00393404	2.05404	2.05404	35.742	1.61761	0.181032	1
16721212	OR51F2	Olfactory receptor, family 51, subfamily F, member 2	ENST00000322110	0.0268037	0.0268037	1.95385	1.95385	11.6899	1.40067	0.479277	1
16898567	GKN2	Gastrokine 2	NM_182536	0.0357134	0.0357134	1.93535	1.93535	9.70058	1.36115	0.561265	1
17091932	CBWD1	COBW domain containing 1	NM_018491	0.00996034	0.00996034	1.89753	1.89753	21.2463	1.28101	0.241173	1
16979330	FLJ14186	Uncharacterized LOC401149	NR_037596	0.00903048	0.00903048	1.89212	1.89212	22.4772	1.26961	0.225938	1
16810972	SNORD18B	Small nucleolar RNA, C/D box 18B	NR_002442	0.0054852	0.0054852	1.88865	1.88865	29.7664	1.2623	0.169627	1
17118419	CDC14B	Cell division cycle 14B	ENST00000481149	0.00759399	0.00759399	1.86621	1.86621	24.8066	1.2153	0.195963	1
17046586	SNORA22	Small nucleolar RNA, H/ACA box 22	NR_002961	0.0327419	0.0327419	1.86355	1.86355	10.2726	1.20974	0.471057	1
16774764	RNY3P2	RNA, Ro-associated Y3 pseudogene 2	ENST00000362918	0.00917447	0.00917447	1.8395	1.8395	22.2744	1.15979	0.208274	1
16888454	FSIP2	Fibrous sheath interacting protein 2	NM_173651	0.0274427	0.0274427	1.8231	1.8231	11.5154	1.12595	0.39111	1
17093018	EQTN	Equatorin, sperm acrosome associated	NM_020641	0.00143389	0.00143389	1.81232	1.81232	61.367	1.10383	0.071949	1
16761514	TAS2R19	Taste receptor, type 2, member 19	ENST00000390673	0.0413512	0.0413512	1.80937	1.80937	8.79088	1.0978	0.499517	1
16760825	CLEC4C	C-type lectin domain family 4, member C	BC114338	0.0122955	0.0122955	1.7908	1.7908	18.7974	1.05992	0.225547	1
17111801	IGBP1-AS1	IGBP1 antisense RNA 1	ENST00000366397	0.0423266	0.0423266	1.76075	1.76075	8.65231	0.99924	0.461953	1
16665507	FOXD3	Forkhead box D3	NM_012183	0.00367629	0.00367629	1.75292	1.75292	37.087	0.98356	0.106081	1
17115154	TREX2	Three prime repair exonuclease 2	NM_080701	0.0422422	0.0422422	1.73375	1.73375	8.66412	0.945401	0.436467	1

16942369	SNTN	Sentan, cilia apical structure protein	ENST00000343837	0.0333035	0.0333035	1.72339	1.72339	10.1586	0.92493	0.364197	1
17117114	BCORP1	BCL6 corepressor pseudogene 1	NR_033732	0.0163674	0.0163674	1.72119	1.72119	15.86	0.920585	0.232177	1
16788669	SNORD114-17	Small nucleolar RNA, C/D box 114-17	NR_003210	0.0290858	0.0290858	1.71756	1.71756	11.0938	0.91344	0.329352	1
16830343	SLC2A4	Solute carrier family 2 (facilitated glucose transporter), member	NM_001042	0.00487364	0.00487364	1.71049	1.71049	31.7786	0.899565	0.113229	1
17112362	MIR4328	MicroRNA 4328	NR_036258	0.0301389	0.0301389	1.70784	1.70784	10.8419	0.89438	0.329971	1
16865060	MIR517A	MicroRNA 517a	NR_030201	0.00189589	0.00189589	1.702	1.702	52.9378	0.882966	0.066717	1
16755223	KRT19P2	Keratin 19 pseudogene 2	NR_036685	0.0430275	0.0430275	1.66399	1.66399	8.5557	0.80955	0.378485	1
17108740	P2RY8	Purinergic receptor P2Y, G-protein coupled, 8	ENST00000460672	0.0237277	0.0237277	1.66232	1.66232	12.6261	0.806379	0.255464	1
17046422	ZNF735	Zinc finger protein 735	NM_001159524	0.010731	0.010731	1.65518	1.65518	20.3501	0.792771	0.155827	1
16803618	MIR184	MicroRNA 184	NR_029705	0.0196718	0.0196718	1.65475	1.65475	14.183	0.791958	0.223354	1
16878888	MIR558	MicroRNA 558	NR_030285	0.025101	0.025101	1.65445	1.65445	12.1868	0.791372	0.259747	1
16814628	TPSD1	Tryptase delta 1	NM_012217	0.0319127	0.0319127	1.65202	1.65202	10.4463	0.786757	0.301258	1
16941704	ITIH1	Inter-alpha-trypsin inhibitor heavy chain 1	NM_001166434	0.0102624	0.0102624	1.65014	1.65014	20.883	0.7832	0.150017	1
16812373	LOC100133746	Uncharacterized LOC100133746	ENST00000548231	0.0197171	0.0197171	1.64507	1.64507	14.163	0.773611	0.218488	1
16934881	SOX10	SRY (sex determining region Y)-box 10	NM_006941	0.039498	0.039498	1.64284	1.64284	9.06821	0.769392	0.33938	1
17005790	HIST2H4B	Histone cluster 2, H4b	ENST00000354348	0.0095927	0.0095927	1.64185	1.64185	21.7115	0.76753	0.141405	1
16731054	MIR34B	MicroRNA 34b	NR_029839	0.0384154	0.0384154	1.63941	1.63941	9.23947	0.762938	0.330295	1
16831527	CCDC144A	Coiled-coil domain containing 144A	ENST00000443444	0.0402288	0.0402288	1.63901	1.63901	8.95654	0.762174	0.340388	1
16895337	POMC	Proopiomelanocortin	NM_001035256	0.0323654	0.0323654	1.63019	1.63019	10.3506	0.745614	0.288143	1
16659794	CLCNKA	Chloride channel, voltage- sensitive Ka	NM_004070	0.031585	0.031585	1.62784	1.62784	10.5169	0.741219	0.281916	1
16705992	MIR4676	MicroRNA 4676	NR_039823	0.034681	0.034681	1.61913	1.61913	9.89091	0.725003	0.2932	1
17107371	SRD5A1P1	Steroid-5-alpha-reductase, alpha polypeptide 1 pseudogene 1	NR_028597	0.0458398	0.0458398	1.6169	1.6169	8.19069	0.720845	0.352031	1
16703659	MAP3K8	Mitogen-activated protein kinase kinase kinase 8	AB209539	0.00793012	0.00793012	1.59747	1.59747	24.2051	0.68504	0.113206	1

16747917	FAM66C	Family with sequence similarity 66, member C	NR_026788	0.0333728	0.0333728	1.59684	1.59684	10.1447	0.683887	0.269653	1
17095020	NMRK1	Nicotinamide riboside kinase 1	NR_023352	0.0252456	0.0252456	1.58741	1.58741	12.1426	0.666679	0.219616	1
16785083	HIF1A	Hypoxia inducible factor 1, alpha subunit (basic helix-loop	ENST00000554177	0.0329583	0.0329583	1.58611	1.58611	10.2283	0.664318	0.259796	1
16927749	IGLV1-44	Immunoglobulin lambda variable 1-44	ENST00000390297	0.023334	0.023334	1.58288	1.58288	12.7592	0.658459	0.206427	1
16909760	ASB18	Ankyrin repeat and SOCS box containing 18	NM_212556	0.0360273	0.0360273	1.579	1.579	9.64435	0.651438	0.270184	1
16852921	LOC284294	Uncharacterized LOC284294	NR_033881	0.00617287	0.00617287	1.57756	1.57756	27.8715	0.648843	0.093119	1
16748275	CLEC9A	C-type lectin domain family 9, member A	NM_207345	0.0166945	0.0166945	1.57568	1.57568	15.672	0.645455	0.164741	1
16862815	ZNF575	Zinc finger protein 575	NM_174945	0.0448975	0.0448975	1.57563	1.57563	8.30913	0.645356	0.310673	1
16969155	MIR3684	MicroRNA 3684	NR_037455	0.0330968	0.0330968	1.56999	1.56999	10.2002	0.635232	0.249106	1
16873160	ZFP112	Zinc finger protein 112 homolog	NM_001083335	0.03461	0.03461	1.56926	1.56926	9.90432	0.633923	0.256018	1
16678946	LOC100506810	Uncharacterized LOC100506810	NR_038856	0.00234529	0.00234529	1.5632	1.5632	47.2635	0.623077	0.052732	1
17014798	SMOC2	SPARC related modular calcium binding 2	NM_022138	0.0471266	0.0471266	1.56271	1.56271	8.03474	0.622195	0.309753	1
16811577	LOC283731	Uncharacterized LOC283731	NR_027073	0.0212716	0.0212716	1.56145	1.56145	13.5157	0.619947	0.183474	1
16803469	DNAJA4	DnaJ (Hsp40) homolog, subfamily A, member 4	NM_018602	0.0271638	0.0271638	1.56123	1.56123	11.5908	0.619556	0.213809	1
16701634	OR14I1	Olfactory receptor, family 14, subfamily I, member 1	ENST00000342623	0.010847	0.010847	1.56073	1.56073	20.2235	0.618673	0.122367	1
16954705	LINC00696	Long intergenic non-protein coding RNA 696	NR_027331	0.0190837	0.0190837	1.55961	1.55961	14.4492	0.616686	0.170718	1
16958638	KLF15	Kruppel-like factor 15	NM_014079	0.00203996	0.00203996	1.55624	1.55624	50.9155	0.610687	0.047977	1
16697272	LOC100288079	Microtubule-associated protein 1 light chain 3 beta	NR_038424	0.039607	0.039607	1.55339	1.55339	9.05136	0.605634	0.267644	1
16673341	RNA5SP65	RNA, 5S ribosomal pseudogene 65	ENST00000363166	0.042946	0.042946	1.54523	1.54523	8.56681	0.591249	0.276065	1
16778677	LINC0033	Long intergenic non-protein coding RNA330	AK056732	0.0313837	0.0313837	1.54379	1.54379	10.5608	0.588709	0.22298	1

16809123	USP50	Ubiquitin specific peptidase 50	NM_203494	0.0100706	0.0100706	1.54204	1.54204	21.1119	0.585633	0.110958	1
16679799	OR2T4	Olfactory receptor, family 2, subfamily T, member 4	NM_001004696	0.0318457	0.0318457	1.54202	1.54202	10.4606	0.585598	0.223924	1
16799776	OIP5-AS1	OIP5 antisense RNA 1	ENST00000558945	0.0347324	0.0347324	1.53624	1.53624	9.88123	0.575486	0.232961	1
16927742	IGLV7-46	Immunoglobulin lambda variable 7-46 (gene/pseudogene)	ENST00000390295	0.0340871	0.0340871	1.53516	1.53516	10.0043	0.573606	0.229344	1
16788731	MIR1193	MicroRNA 1193	NR_036132	0.0164339	0.0164339	1.52923	1.52923	15.8214	0.563296	0.142414	1
16804062	TM6SF1	Transmembrane 6 superfamily member 1	NM_023003	0.00223439	0.00223439	1.52746	1.52746	48.503	0.560234	0.046202	1
16704027	ZNF37A	Zinc finger protein 37A	NM_001007094	0.0152454	0.0152454	1.52651	1.52651	16.5503	0.558585	0.135003	1
16849597	C1QTNF1-AS1	C1QTNF1 antisense RNA 1	NR_040018	0.0433442	0.0433442	1.52573	1.52573	8.51281	0.557226	0.26183	1
16667481	DPYD-AS1	DPYD antisense RNA 1	NR_046590	0.0116713	0.0116713	1.52374	1.52374	19.3792	0.553798	0.114307	1
16725084	OR5A1	Olfactory receptor, family 5, subfamily A, member 1	ENST00000302030	0.00858497	0.00858497	1.52101	1.52101	23.1366	0.549097	0.094931	1
16926043	TFF1	Trefoil factor 1	ENST00000291527	0.0368823	0.0368823	1.51829	1.51829	9.49486	0.544416	0.229352	1
16865498	KIR3DL1	Killer cell immunoglobulin-like receptor, three domains, long cytoplasmic tail	NM_013289	0.040739	0.040739	1.51015	1.51015	8.88038	0.530494	0.238951	1
16858756	GCDH	Glutaryl-CoA dehydrogenase	NM_000159	0.0393276	0.0393276	0.666147	-1.50117	9.09469	0.51525	0.226616	1
16826619	LOC100132339	Uncharacterized LOC100132339	ENST00000559802	0.0432994	0.0432994	0.665876	-1.50178	8.51885	0.516279	0.242417	1
16657895	MMP23B	Matrix metallopeptidase 23B	NM_006983	0.00553606	0.00553606	0.665351	-1.50297	29.6143	0.518284	0.070005	1
16812517	LOC80154	Golgin A2 pseudogene	NR_026811	0.0249706	0.0249706	0.664977	-1.50381	12.2269	0.519717	0.170024	1
17069141	LOC286177	Uncharacterized LOC286177	NR_038874	0.0109349	0.0109349	0.662864	-1.5086	20.1289	0.527856	0.104895	1
16848692	HID1	HID1 domain containing	NM_030630	0.0300424	0.0300424	0.661961	-1.51066	10.8644	0.531363	0.195634	1
17042805	GET4	Golgi to ER traffic protein 4 homolog (S. cerevisiae)	NM_015949	0.0420725	0.0420725	0.659264	-1.51684	8.68794	0.541931	0.24951	1
16686376	ZSWIM5	Zinc finger, SWIM-type containing 5	NM_020883	0.00694559	0.00694559	0.657235	-1.52153	26.0879	0.54998	0.084327	1
17008529	TOMM6	Translocase of outer mitochondrial membrane 6 homolog (yeast)	AJ420506	0.0161474	0.0161474	0.656565	-1.52308	15.9897	0.552657	0.138253	1
16942389	THOC7-AS1	THOC7 antisense RNA 1	ENST00000468961	0.0496952	0.0496952	0.65602	-1.52434	7.74189	0.554838	0.286668	1
16659881	NECAP2	NECAP endocytosis associated 2	NM_018090	0.0152683	0.0152683	0.6554	-1.52579	16.5354	0.557332	0.134821	1

16703758	RNU7-22P	RNA, U7 small nuclear 22 pseudogene	ENST00000516673	0.0441043	0.0441043	0.655306	-1.526	8.4118	0.55771	0.265204	1
17068782	MCM4	Minichromosome maintenance complex component 4	NM_005914	0.0380317	0.0380317	0.650849	-1.53646	9.30193	0.575867	0.247633	1
16920876	LOC100128310	Uncharacterized LOC100128310	AK097866	0.0298676	0.0298676	0.650784	-1.53661	10.9055	0.576132	0.211318	1
16916158	MIR941-1	MicroRNA 941-1	NR_030637	0.0324546	0.0324546	0.649299	-1.54012	10.332	0.582276	0.225427	1
16910854	EBF4	Early B-cell factor 4	NM_001110514	0.0363486	0.0363486	0.648818	-1.54126	9.58756	0.584278	0.243765	1
16780268	LOC100652869	Uncharacterized LOC100652869	XR_171060	0.00617201	0.00617201	0.648237	-1.54265	27.8737	0.586701	0.084194	1
17063975	CTAGE6	CTAGE family, member 6	NM_178561	0.0381603	0.0381603	0.64822	-1.54269	9.2809	0.586772	0.252895	1
16708061	ZFYVE27	Zinc finger, FYVE domain containing 27	NM_144588	0.0459347	0.0459347	0.647782	-1.54373	8.17896	0.588601	0.287861	1
16914117	PABPC1L	Poly(A) binding protein, cytoplasmic 1-like	NM_001124756	0.0426532	0.0426532	0.646879	-1.54588	8.60699	0.592391	0.275307	1
17057897	VOPP1	Vesicular, overexpressed in cancer, prosurvival protein 1	NM_030796	0.0118575	0.0118575	0.64688	-1.54588	19.2008	0.592387	0.123409	1
16671049	LCE2A	Late cornified envelope 2A	NM_178428	0.0158713	0.0158713	0.646083	-1.54779	16.1562	0.595745	0.147496	1
16681127	HES2	Hairy and enhancer of split 2	NM_019089	0.0345635	0.0345635	0.646077	-1.5478	9.91312	0.595768	0.240396	1
16728141	GAL	Galanin/GMAP prepropeptide	ENST00000265643	0.0449885	0.0449885	0.64506	-1.55024	8.29753	0.600076	0.289279	1
16928428	ADRBK2	Adrenergic, beta, receptor kinase 2	NM_005160	0.0292868	0.0292868	0.644519	-1.55154	11.0446	0.602373	0.21816	1
16664438	LINC00853	Long intergenic non-protein coding RNA 853	NR_047498	0.00392191	0.00392191	0.644396	-1.55184	35.8023	0.602895	0.067358	1
17092187	RLN1	Relaxin 1	NM_006911	0.00601878	0.00601878	0.641863	-1.55797	28.2677	0.613754	0.086849	1
16798951	GREM1	Gremlin 1, DAN family BMP antagonist	ENST00000300177	0.0467654	0.0467654	0.641705	-1.55835	8.07785	0.614435	0.304256	1
17118281	LOC100506458	Putative uncharacterized protein LOC65996-like	AK097034	0.039974	0.039974	0.640889	-1.56033	8.99512	0.617963	0.274799	1
16822548	NARFL	Nuclear prelamin A recognition factor-like	NM_022493	0.0279976	0.0279976	0.640866	-1.56039	11.3689	0.618064	0.217458	1
17016363	HIST1H3B	Histone cluster 1, H3b	NM_003537	0.0075093	0.0075093	0.63827	-1.56673	24.9645	0.62939	0.100846	1
16932782	PI4KAP2	Phosphatidylinositol 4-Kinase, catalytic, alpha pseudogene 2	NR_003700	0.0100812	0.0100812	0.637439	-1.56878	21.099	0.633052	0.120015	1

17049667	TRIM56	Tripartite motif containing 56	ENST00000306085	0.0303044	0.0303044	0.636306	-1.57157	10.8035	0.638063	0.236242	1
17004339	PSMG4	Proteasome (prosome, macropain) assembly chaperone 4	AK096543	0.0496349	0.0496349	0.635179	-1.57436	7.7485	0.643074	0.331973	1
16858263	QTRT1	Queuine tRNA-ribosyltransferase 1	NM_031209	0.0424447	0.0424447	0.634868	-1.57513	8.63587	0.644462	0.298505	1
16739317	MTA2	Metastasis associated 1 family, member 2	NM_004739	0.0435419	0.0435419	0.634777	-1.57536	8.48628	0.644872	0.30396	1
17101605	TCEANC	Transcription elongation factor A (SII) N-terminal and central domain containing	NM_152634	0.0225791	0.0225791	0.633589	-1.57831	13.024	0.650197	0.199692	1
17081850	GPR20	G protein-coupled receptor 20	NM_005293	0.0215886	0.0215886	0.633532	-1.57845	13.3924	0.650454	0.194276	1
17117486	LOC387720	Uncharacterized LOC387720	AK127642	0.0166138	0.0166138	0.632389	-1.58131	15.7179	0.655612	0.166844	1
16910645	FAM110A	Family with sequence similarity 110, member A	ENST00000381941	0.0166047	0.0166047	0.631965	-1.58237	15.7231	0.657532	0.167278	1
17104947	SLC16A2	Solute carrier family 16, member 2 (thyroid hormone transporter	NM_006517	0.0311806	0.0311806	0.631733	-1.58295	10.6055	0.658586	0.248395	1
17073290	GPIHBP1	Glycosylphosphatidyl inositol anchored high density lipoprotein	NM_178172	0.00588708	0.00588708	0.630145	-1.58694	28.6186	0.66582	0.093061	1
16864084	CCDC155	Coiled-coil domain containing 155	NM_144688	0.0240895	0.0240895	0.62944	-1.58871	12.5067	0.669053	0.213982	1
16822801	NME3	NME/NM23 nucleoside diphosphate kinase 3	ENST00000219302	0.0194712	0.0194712	0.627934	-1.59252	14.2725	0.675997	0.189455	1
16857242	LOC100131094	Uncharacterized LOC100131094	NM_001242901	0.0406088	0.0406088	0.626812	-1.59537	8.89968	0.6812	0.306168	1
16757873	SIRT4	Sirtuin 4	NM_012240	0.0206501	0.0206501	0.625628	-1.59839	13.7657	0.686728	0.199547	1
16753962	MIR3913-1	MicroRNA 3913-1	NR_037475	0.0485542	0.0485542	0.623832	-1.603	7.86908	0.695175	0.353371	1
17013520	SASH1	SAM and SH3 domain containing 1	NM_015278	0.049728	0.049728	0.621149	-1.60992	7.7383	0.707932	0.365937	1
16806538	GOLGA8R	Golgin A8 family, member R	ENST00000544495	0.032288	0.032288	0.621122	-1.60999	10.3668	0.708062	0.273203	1
16729290	TSKU	Tsukushi, small leucine rich proteoglycan	NM_001258210	0.00953557	0.00953557	0.618969	-1.61559	21.7862	0.718422	0.131904	1
16990146	WDR55	WD repeat domain 55	ENST00000358337	0.0256844	0.0256844	0.61618	-1.6229	12.011	0.732015	0.243782	1
16715223	C10orf105	Chromosome 10 open reading frame 105	NM_001164375	0.0457697	0.0457697	0.615783	-1.62395	8.19937	0.733965	0.358059	1
17110700	KCND1	Potassium voltage-gated channel, Shal-related subfamily D, member 1	NM_004979	0.00600062	0.00600062	0.613899	-1.62893	28.3154	0.74327	0.104999	1
16857630	PNPLA6	Patatin-like phospholipase domain containing 6	NM_001166111	0.00386235	0.00386235	0.613384	-1.6303	36.1026	0.74583	0.082635	1

16774623	LRCH1	Leucine-rich repeats and calponin homology (CH) domain containing 1	ENST00000311191	0.00434677	0.00434677	0.613272	-1.6306	33.8429	0.746387	0.088218	1
16795755	TTC7B	Tetratricopeptide repeat domain 7B	NM_001010854	0.0133899	0.0133899	0.61314	-1.63095	17.8772	0.747042	0.16715	1
17033279	MICA	MHC class I polypeptide-related sequence A	NR_036523	0.0103894	0.0103894	0.610552	-1.63786	20.735	0.76002	0.146616	1
16855673	BCL2	B-cell CLL/lymphoma 2	ENST00000398117	0.0204666	0.0204666	0.60862	-1.64306	13.8417	0.769815	0.222462	1
17060983	POLR2J	Polymerase (RNA) II (DNA directed) polypeptideJ, 13.3kDa	NM_006234	0.0433766	0.0433766	0.603216	-1.65778	8.50846	0.797715	0.375022	1
16741549	ALG1L9P	Asparagine-linked glycosylation 1-like 9, pseudogene	NR_073388	0.0140843	0.0140843	0.60178	-1.66174	17.35	0.805255	0.18565	1
16807195	RASGRP1	RAS guanyl releasing protein 1 (calcium and DAG-regulated)	NM_005739	0.0479793	0.0479793	0.598616	-1.67052	7.9349	0.822058	0.414401	1
16662108	TSSK3	Testis-specific serine Kinase 3	NM_052841	0.021244	0.021244	0.59463	-1.68172	13.5266	0.843605	0.249466	1
17003077	RNF44	Ring finger protein 44	NM_014901	4.37E-05	4.37E-05	0.592431	-1.68796	367.338	0.855672	0.009318	1
17049702	ZNHIT1	Zinc finger, HIT-type containing 1	NM_006349	0.0394297	0.0394297	0.592168	-1.68871	9.0788	0.857124	0.377638	1
16797285	BRF1	RNA polymerase III transcription initiation factor 90	ENST00000546474	0.0421155	0.0421155	0.590962	-1.69216	8.6819	0.86381	0.397982	1
16867657	PSPN	Persephin	NM_004158	0.0382368	0.0382368	0.589907	-1.69518	9.26843	0.869686	0.375333	1
17041421	ZBTB9	Zinc finger and BTB domain containing 9	ENST00000395064	0.0438848	0.0438848	0.587237	-1.70289	8.44069	0.884702	0.419256	1
17032957	HLA-L	Major histocompatibility complex, class I, L (pseudogene)	ENST00000491405	0.0169509	0.0169509	0.585126	-1.70903	15.5285	0.896712	0.230984	1
16842070	TOP3A	Topoisomerase (DNA) III alpha	NM_004618	0.0292841	0.0292841	0.584694	-1.7103	11.0453	0.899185	0.325635	1
16795567	KCNK10	Potassium channel, subfamily K, member 10	NM_021161	0.00668772	0.00668772	0.581317	-1.72023	26.6486	0.918699	0.137898	1
16946016	PCCB	Propionyl CoA carboxylase, beta polypeptide	NM_000532	0.0413342	0.0413342	0.580743	-1.72193	8.79333	0.922048	0.419431	1
16817212	RNA5SP405	RNA, 5S ribosomal pseudogene 405	ENST00000363059	0.0341409	0.0341409	0.578528	-1.72853	9.99393	0.935064	0.374253	1
16848961	TRIM65	Tripartite motif containing 65	NM_173547	0.0321262	0.0321262	0.578186	-1.72955	10.4009	0.937083	0.360384	1
16748304	GABARAPL1	GABA(A) receptor-associated protein like 1	ENST00000266458	0.0218829	0.0218829	0.576816	-1.73365	13.2803	0.945216	0.284697	1
16995888	LOC648987	Uncharacterized LOC648987	ENST00000503152	0.03522	0.03522	0.576709	-1.73398	9.7905	0.945855	0.386438	1

16859874	UPF1	UPF1 regulator of nonsense transcripts homolog	NM_002911	0.0407804	0.0407804	0.572561	-1.74654	8.87427	0.970827	0.437592	1
17057813	COBL	Cordon-bleu WH2 repeat protein	NM_015198	0.0257134	0.0257134	0.571697	-1.74918	12.0024	0.976094	0.325301	1
16916221	TCEA2	Transcription elongation factor A (SII), 2	BC098585	0.00983256	0.00983256	0.569842	-1.75487	21.4051	0.987474	0.184531	1
16814619	TPSAB1	Tryptase alpha/beta 1	NM_003294	0.0458649	0.0458649	0.567337	-1.76262	8.18758	1.003	0.490012	1
16902679	LOC389033	Placenta-specific 9 pseudogene	NR_026740	0.0474685	0.0474685	0.566148	-1.76632	7.99438	1.01044	0.505577	1
16894824	OSR1	Odd-skipped related 1	NM_145260	0.00987536	0.00987536	0.558694	-1.78989	21.3516	1.05807	0.19822	1
17111464	FGD1	FYVE, RhoGEF and PH domain containing 1	NM_004463	0.0119947	0.0119947	0.558614	-1.79014	19.0721	1.05859	0.222019	1
16865693	NAT14	N-acetyltransferase 14 (GCN5-related, putative)	NM_020378	0.0282187	0.0282187	0.557613	-1.79336	11.3117	1.06512	0.376645	1
16843376	NLE1	Notchless homolog 1 (Drosophila)	NM_001014445	0.0354797	0.0354797	0.552428	-1.81019	9.74294	1.09947	0.45139	1
16723100	MIR610	MicroRNA 610	NR_030341	0.00238537	0.00238537	0.552107	-1.81124	46.8369	1.10162	0.094082	1
16722295	RNA5SP332	RNA, 5S ribosomal pseudogene 332	ENST00000391063	0.0278526	0.0278526	0.546166	-1.83095	11.4068	1.14212	0.400505	1
16711484	IL15RA	Interleukin 15 receptor, alpha	NM_001243539	0.0291015	0.0291015	0.545693	-1.83253	11.0899	1.14539	0.413128	1
16808340	CATSPER2P1	Cation channel, sperm associated 2 pseudogene 1	NR_002318	0.040096	0.040096	0.544994	-1.83488	8.9766	1.15024	0.512551	1
17000121	LOC340073	Uncharacterized LOC340073	NR_037895	0.00807635	0.00807635	0.544351	-1.83705	23.9553	1.15472	0.192813	1
16758186	PSMD9	Proteasome (prosome, macropain) 26S subunit, non-ATPase, 9	NM_002813	0.0448884	0.0448884	0.534256	-1.87176	8.31029	1.2269	0.590545	1
16717706	POLL	Polymerase (DNA directed), lambda	NM_001174084	0.0241437	0.0241437	0.533809	-1.87333	12.4891	1.23018	0.394001	1
16855049	TCEB3C	Transcription elongation factor B polypeptide 3C (elongin A3)	NM_145653	0.020991	0.020991	0.53241	-1.87825	13.6272	1.24048	0.364119	1
17047138	GTF2IRD1	GTF2I repeat domain containing 1	NM_016328	0.0474343	0.0474343	0.531654	-1.88092	7.99839	1.24609	0.623169	1
16891225	TUBA4B	Tubulin, alpha 4b (pseudogene)	NR_003063	0.0327691	0.0327691	0.524633	-1.90609	10.267	1.29908	0.50612	1
16673883	C1orf105	Chromosome 1 open reading frame 105	ENST00000367727	0.00242651	0.00242651	0.522491	-1.91391	46.4101	1.31561	0.11339	1
16952244	ACAA1	Acetyl-CoA acyltransferase 1	NM_001607	0.0418213	0.0418213	0.514133	-1.94502	8.72349	1.38179	0.633593	1
16812083	LOC91450	Uncharacterized LOC91450	NR_026998	0.00067377	0.00067377	0.490577	-2.03842	91.0427	1.58348	0.069571	1
17056506	RP9P	Retinitis pigmentosa 9 pseudogene	NR_003500	0.00704435	0.00704435	0.489133	-2.04443	25.8814	1.59661	0.246759	1

17046559	INTS4L2	Integrator complex subunit 4-like 2	NR_027392	0.0391764	0.0391764	0.483515	-2.06819	9.11834	1.64861	0.723206	1
17035366	LOC100996357	Uncharacterized LOC100996357	ENST00000436256	0.0047095	0.0047095	0.482577	-2.07221	32.3844	1.65744	0.20472	1
17052679	IL23A	Interleukin 23, alpha subunit p19	AY532914	0.0291177	0.0291177	0.481004	-2.07899	11.0859	1.67232	0.603403	1
17108719	ASMTL	Acetylserotonin O-methyltransferase-like	ENST00000463763	0.00620783	0.00620783	0.480388	-2.08165	27.7837	1.67818	0.241606	1
16782207	LRP10	Low density lipoprotein receptor-related protein 10	ENST00000359591	0.0141519	0.0141519	0.477584	-2.09387	17.3007	1.70509	0.394225	1
16862484	CCDC97	Coiled-coil domain containing 97	NM_052848	0.0355213	0.0355213	0.457644	-2.18511	9.73536	1.90757	0.78377	1
16789743	CRIP1	Cysteine-rich protein 1 (intestinal)	NM_001311	0.0413088	0.0413088	0.436936	-2.28866	8.79701	2.14027	0.973179	1
16771801	HCAR3	Hydroxycarboxylic acid receptor 3	NM_006018	0.0339516	0.0339516	0.429125	-2.33032	10.0306	2.23454	0.891089	1
17051157	RNU7-27P	RNA, U7 small nuclear 27 pseudogene	ENST00000459281	0.00793745	0.00793745	0.413836	-2.41642	24.1925	2.43029	0.401826	1
16691129	AP4B1	Adaptor-related protein complex 4, beta 1 subunit	NM_001253852	0.0497166	0.0497166	0.356928	-2.80168	7.73954	3.3136	1.71256	1
16840284	C1QBP	Complement component 1, q subcomponent binding protein	NM_001212	0.00200878	0.00200878	0.199122	-5.02205	51.3347	8.13131	0.633592	1

Table S2. Functional categorization of genes that were affected after knockdown of C1QBP in MDA-MB-231 breast cancer cells

Function	Gene ID	Gene name	Gene EntrezID
Transcription elongation factor	TCEANC	Transcription elongation factor A (SII) N-terminal and central d	170082
	TCEB3C	Transcription elongation factor B polypeptide 3C (elongin A3)	728929
	TCEA2	Transcription elongation factor A (SII), 2	6919
Metal-ion binding	FGD1	FYVE, RhoGEF and PH domain containing 1	2245
	ZBTB9	Zinc finger and BTB domain containing 9	221504
	QTRT1	Queuine tRNA-ribosyltransferase 1	81890
	ZFP112	Zinc finger protein 112 homolog	7771
	TRIM65	Tripartite motif containing 65	201292
	OSR1	Odd-skipped related 1	130497
	RASGRP1	RAS guanyl releasing protein 1 (calcium and DAG-regulated)	10125
	ZNF735	Zinc finger protein 735	168417
	TCEA2	Transcription elongation factor A (SII), 2	6919
	DNAJA4	DnaJ (Hsp40) homolog, subfamily A, member 4	55466
	ZNF575	Zinc finger protein 575	284346
	CRIP1	Cysteine-rich protein 1 (intestinal)	1396
	BRF1	RNA polymerase III transcription initiation factor 90	2972
	MTA2	Metastasis associated 1 family, member 2	9219
	SIRT4	Sirtuin 4	23409
	KLF15	Kruppel-like factor 15	28999

	ZNF37A	Zinc finger protein 37A	7587
	ZSWIM5	Zinc finger, SWIM-type containing 5	57643
	TRIM56	Tripartite motif containing 56	81844
	RNF44	Ring finger protein 44	22838
	EBF4	Early B-cell factor 4	57593
	ZFYVE27	Zinc finger, FYVE domain containing 27	118813
	MMP23B	Matrix metallopeptidase 23B	8510
	TOP3A	Topoisomerase (DNA) III alpha	7156
	ZNHIT1	Zinc finger, HIT-type containing 1	10467
	UPF1	UPF1 regulator of nonsense transcripts homolog	5976
	MAP3K8	Mitogen-activated protein kinase kinase kinase 8	1326
	POLL	Polymerase (DNA directed), lambda	27343
	KCND1	Potassium voltage-gated channel, Shal-related subfamily, member 1	3750
	NME3	NME/NM23 nucleoside diphosphate kinase 3	4832
	KCNK10	Potassium channel, subfamily K, member 10	54207
	SMOC2	SPARC related modular calcium binding 2	64094
	ITIH1	Inter-alpha-trypsin inhibitor heavy chain 1	3697
	TREX2	Three prime repair exonuclease 2	11219
	SNTN	Sentan, cilia apical structure protein	132203
Neurogenesis	PSPN	Persephin	5623
	OSR1	Odd-skipped related 1	130497
	BCL2	B-cell CLL/lymphoma 2	596
	GREM1	Gremlin 1, DAN family BMP antagonist	26585
	LCE2A	Late cornified envelope 2A	353139
Regulation of transcription	POLL	Polymerase (DNA directed), lambda	27343
	SOX10	SRY (sex determining region Y)- box 10	6663
	ZBTB9	Zinc finger and BTB domain containing 9	221504

MTA2	Metastasis associated 1 family, member 2	9219
KLF15	Kruppel-like factor 15	28999
ZFP112	Zinc finger protein 112 homolog	7771
MCM4	Minichromosome maintenance complex component 4	4173
ZNF37A	Zinc finger protein 37A	7587
HIST2H4B	Histone cluster 2, H4b	8370
HIF1A	Hypoxia inducible factor 1, alpha subunit (basic helix-loop	3091
EBF4	Early B-cell factor 4	57593
GTF2IRD1	GTF2I repeat domain containing 1	9569
HES2	Hairy and enhancer of split 2	54626
TOP3A	Topoisomerase (DNA) III alpha	7156
HIST1H3B	Histone cluster 1, H3b	126961
ZNF735	Zinc finger protein 735	168417
TCEA2	Transcription elongation factor A (SII), 2	6919
ZNF575	Zinc finger protein 575	284346
NAT14	N-acetyltransferase 14 (GCN5- related, putative)	57106
FOXD3	Forkhead box D3	27022
TCEANC	Transcription elongation factor A (SII) N-terminal and central d	170082
POLR2J	Polymerase (RNA) II (DNA directed) polypeptide J, 13.3kDa	5439
BRF1	RNA polymerase III transcription initiation factor 90	2972
UPF1	UPF1 regulator of nonsense transcripts homolog	5976
TCEB3C	Transcription elongation factor B polypeptide 3C (elongin A3)	728929
BCL2	B-cell CLL/lymphoma 2	596
PSMD9	Proteasome (prosome, macropain) 26S subunit, non- ATPase, 9	5715
VOPP1	Vesicular, overexpressed in cancer, prosurvival protein 1	81552

	CDC14B	Cell division cycle 14B	8555
	QTRT1	Queuine tRNA-ribosyltransferase 1	81890
	WDR55	WD repeat domain 55	54853
	OSR1	Odd-skipped related 1	130497
	IL15RA	Interleukin 15 receptor, alpha	3601
	TREX2	Three prime repair exonuclease 2	11219
	NLE1	Notchless homolog 1 (Drosophila)	54475
Immune response	MICA	MHC class I polypeptide-related sequence A	4276
	IL23A	Interleukin 23, alpha subunit p19	51561
	ITIH1	Inter-alpha-trypsin inhibitor heavy chain 1	3697
	BCL2	B-cell CLL/lymphoma 2	596
	KIR3DL1	Killer cell immunoglobulin-like receptor, three domains, long c	3811
	POLL	Polymerase (DNA directed), lambda	27343
	IGLV1-44	Immunoglobulin lambda variable 1-44	3538
	C1QBP	Complement component 1, q subcomponent binding protein	708
	HLA-L	Major histocompatibility complex, class I, L (pseudogene)	3139
	CLEC4C	C-type lectin domain family 4, member C	170482
	TPSAB1	Tryptase alpha/beta 1	7177
	GAL	Galanin/GMAP prepropeptide	51083
	HIF1A	Hypoxia inducible factor 1, alpha subunit (basic helix-loop	3091
	HPR	Haptoglobin-related protein	3250
Hormone activity	RLN1	Relaxin 1	6013
	GAL	Galanin/GMAP prepropeptide	51083
	POMC	Proopiomelanocortin	5443
	MMP23B	Matrix metallopeptidase 23B	8510
Phosphorylation activity	CDC14B	Cell division cycle 14B	8555
	BCL2	B-cell CLL/lymphoma 2	596
	CCDC155	Coiled-coil domain containing 155	147872

	TPTE	Transmembrane phosphatase with tensin homology	7179
	MAP3K8	Mitogen-activated protein kinase kinase kinase 8	1326
	PI4KAP2	Phosphatidylinositol 4-kinase, catalytic, alpha pseudogene 2	375133
	ADRBK2	Adrenergic, beta, receptor kinase 2	157
	TSSK3	Testis-specific serine kinase 3	81629
Regulation of cell motion	HIF1A	Hypoxia inducible factor 1, alpha subunit (basic helix-loop	3091
	BCL2	B-cell CLL/lymphoma 2	596
	GREM1	Gremlin 1, DAN family BMP antagonist	26585
Enzymatic activity	TPSAB1	Tryptase alpha/beta 1	7177
	TPSD1	Tryptase delta 1	23430
	HPR	Haptoglobin-related protein	3250
	MMP23B	Matrix metallopeptidase 23B	8510
	USP50	Ubiquitin specific peptidase 50	373509
	ASB18	Ankyrin repeat and SOCS box containing 18	401036
	PSMD9	Proteasome (prosome, macropain) 26S subunit, non- ATPase, 9	5715
	CDC14B	Cell division cycle 14B	8555
	TREX2	Three prime repair exonuclease 2	11219
	PNPLA6	Patatin-like phospholipase domain containing 6	10908
	TPTE	Transmembrane phosphatase with tensin homology	7179
Homeostasis	HIF1A	Hypoxia inducible factor 1, alpha subunit (basic helix-loop	3091
	BCL2	B-cell CLL/lymphoma 2	596
	NARFL	Nuclear prelamin A recognition factor-like	64428
	SLC2A4	Solute carrier family 2 (facilitated glucose transporter), member	6517
	HPR	Haptoglobin-related protein	3250
	TFF1	Trefoil factor 1	7031

Stress response	POLL	Polymerase (DNA directed), lambda	27343
	MICA	MHC class I polypeptide-related sequence A	4276
	HIF1A	Hypoxia inducible factor 1, alpha subunit (basic helix-loop	3091
	UPF1	UPF1 regulator of nonsense transcripts homolog	5976
	BCL2	B-cell CLL/lymphoma 2	596
	TREX2	Three prime repair exonuclease 2	11219
	TOP3A	Topoisomerase (DNA) III alpha	7156
	MCM4	Minichromosome maintenance complex component 4	4173
Response to hormone stimulus	BCL2	B-cell CLL/lymphoma 2	596
	TFF1	Trefoil factor 1	7031
	GAL	Galanin/GMAP prepropeptide	51083
Sensory perception	OR2T4	Olfactory receptor, family 2, subfamily T, member 4	127074
	OR14I1	Olfactory receptor, family 14, subfamily I, member 1	401994
	ADRBK2	Adrenergic, beta, receptor kinase 2	157
	OR51F2	Olfactory receptor, family 51, subfamily F, member 2	119694
	OR5A1	Olfactory receptor, family 5, subfamily A, member 1	219982
	TAS2R19	Taste receptor, type 2, member 19	259294
	TAS2R31	Taste receptor, type 2, member 31	259290
	P2RY8	Purinergic receptor P2Y, G- protein coupled, 8	286530
	GPR20	G protein-coupled receptor 20	2843
	POMC	Proopiomelanocortin	5443
	LRP10	Low density lipoprotein receptor- related protein 10	26020
	CLEC9A	C-type lectin domain family 9, member A	283420
	IL15RA	Interleukin 15 receptor, alpha	3601

	KIR3DL1	Killer cell immunoglobulin-like receptor, three domains, long c	3811
	GAL	Galanin/GMAP prepropeptide	51083
Chromatin organization	MTA2	Metastasis associated 1 family, member 2	9219
	HIST1H3B	Histone cluster 1, H3b	126961
	HIST2H4B	Histone cluster 2, H4b	8370
Voltage-gated ion channel activity	KCND1	Potassium voltage-gated channel, Shal-related subfamily, member 1	3750
	CLCNKA	Chloride channel, voltage- sensitive Ka	1187
	KCNK10	Potassium channel, subfamily K, member 10	54207
	TPTE	Transmembrane phosphatase with tensin homology	7179
	SLC16A2	Solute carrier family 16, member 2 (thyroid hormone transporter	6567
	SLC2A4	Solute carrier family 2 (facilitated glucose transporter), member	6517
	TOMM6	Translocase of outer mitochondrial membrane 6 homolog (yeast)	100188893
	NECAP2	NECAP endocytosis associated 2	55707
	AP4B1	Adaptor-related protein complex 4, beta 1 subunit	10717
Cell death	FGD1	FYVE, RhoGEF and PH domain containing 1	2245
	MICA	MHC class I polypeptide-related sequence A	4276
	NME3	NME/NM23 nucleoside diphosphate kinase 3	4832
	ZFYVE27	Zinc finger, FYVE domain containing 27	118813
	BCL2	B-cell CLL/lymphoma 2	596
	GREM1	Gremlin 1, DAN family BMP antagonist	26585
	PNPLA6	Patatin-like phospholipase domain containing 6	10908
	GAL	Galanin/GMAP prepropeptide	51083
	RASGRP1	RAS guanyl releasing protein 1 (calcium and DAG-regulated)	10125

	MAP3K8	Mitogen-activated protein kinase kinase kinase 8	1326
Cytoskeleton	GABARAPL1	GABA(A) receptor-associated protein like 1	23766
	TREX2	Three prime repair exonuclease 2	11219
	TUBA4B	Tubulin, alpha 4b (pseudogene)	80086
	FAM110A	Family with sequence similarity 110, member A	83541
Kinase activity and nucleotide binding	MAP3K8	Mitogen-activated protein kinase kinase kinase 8	1326
	ADRBK2	Adrenergic, beta, receptor kinase 2	157
	TSSK3	Testis-specific serine kinase 3	81629
	CBWD1	COBW domain containing 1	55871
	UPF1	UPF1 regulator of nonsense transcripts homolog	5976
	MCM4	Minichromosome maintenance complex component 4	4173
	BCL2	B-cell CLL/lymphoma 2	596
	PI4KAP2	Phosphatidylinositol 4-kinase, catalytic, alpha pseudogene 2	375133
	GCDH	Glutaryl-CoA dehydrogenase	2639
	NME3	NME/NM23 nucleoside diphosphate kinase 3	4832
	TOP3A	Topoisomerase (DNA) III alpha	7156
	PCCB	Propionyl CoA carboxylase, beta polypeptide	5096
	SIRT4	Sirtuin 4	23409
	RAB44	RAB44, member RAS oncogene family	401258
	TUBA4B	Tubulin, alpha 4b (pseudogene)	80086
	PABPC1L	Poly(A) binding protein, cytoplasmic 1-like	80336
Cell cycle	UPF1	UPF1 regulator of nonsense transcripts homolog	5976
	BCL2	B-cell CLL/lymphoma 2	596
	MAP3K8	Mitogen-activated protein kinase kinase kinase 8	1326
	TREX2	Three prime repair exonuclease 2	11219

	TOP3A	Topoisomerase (DNA) III alpha	7156
	PSMD9	Proteasome (prosome, macropain) 26S subunit, non- ATPase, 9	5715
Catabolic processes	MMP23B	Matrix metallopeptidase 23B	8510
	TPSAB1	Tryptase alpha/beta 1	7177
	TPSD1	Tryptase delta 1	23430
	USP50	Ubiquitin specific peptidase 50	373509
	HPR	Haptoglobin-related protein	3250
	ASB18	Ankyrin repeat and SOCS box containing 18	401036
	PSMD9	Proteasome (prosome, macropain) 26S subunit, non- ATPase, 9	5715
	UPF1	UPF1 regulator of nonsense transcripts homolog	5976
Protein transport	TOMM6	Translocase of outer mitochondrial membrane 6 homolog (yeast)	100188893
	NECAP2	NECAP endocytosis associated 2	55707
	AP4B1	Adaptor-related protein complex 4, beta 1 subunit	10717
	RAB44	RAB44, member RAS oncogene family	401258

Table S3. Full list of signalling pathways identified by Partek Software

Pathway Name	Enrichment Score	Genes
Fatty acid degradation	3.51594	ACAA1, GCDH
Valine, leucine and isoleucine degradation	3.51594	ACAA1, PCCB
Taste transduction	3.209	TAS2R19, TAS2R31
Adipocytokine signaling pathway	2.67827	POMC, SLC2A4
Non-homologous end-joining	2.56127	POLL
Olfactory transduction	2.31763	ADRBK2, OR2T4, OR5A1, OR14I1, OR51F
mRNA surveillance pathway	2.22979	PABPC1L, UPF1
Biosynthesis of unsaturated fatty acids	2.10542	ACAA1
T cell receptor signaling pathway	2.01024	MAP3K8, RASGRP1
Pyrimidine metabolism	1.99475	NME3, POLR2J
Glyoxylate and dicarboxylate metabolism	1.98074	PCCB
HIF-1 signaling pathway	1.97945	BCL2, HIF1A
alpha-Linolenic acid metabolism	1.94286	ACAA1
Nicotinate and nicotinamide metabolism	1.83835	NMRK1
Homologous recombination	1.83835	TOP3A
Cell cycle	1.73155	CDC14B, MCM4
Propanoate metabolism	1.71654	PCCB
RNA polymerase	1.71654	POLR2J
Regulation of autophagy	1.66176	GABARAPL1
African trypanosomiasis	1.66176	HPR
Base excision repair	1.66176	POLL
Natural killer cell mediated cytotoxicity	1.64724	KIR3DL1, MICA
FoxO signaling pathway	1.62423	GABARAPL1, SLC2A4
DNA replication	1.61043	MCM4
Systemic lupus erythematosus	1.59054	HIST1H3B, HIST2H4B
Tryptophan metabolism	1.5167	GCDH
Graft-versus-host disease	1.45308	KIR3DL1
Basal transcription factors	1.4134	GTF2IRD1
Jak-STAT signaling pathway	1.37955	IL15RA, IL23A
Fatty acid metabolism	1.35751	ACAA1
Type II diabetes mellitus	1.35751	SLC2A4
Lysine degradation	1.33977	GCDH
Intestinal immune network for IgA production	1.33977	IL15RA
RNA transport	1.30902	PABPC1L, UPF1
Amyotrophic lateral sclerosis (ALS)	1.30551	BCL2
Fanconi anemia pathway	1.27279	TOP3A
Purine metabolism	1.26732	NME3, POLR2J
Tuberculosis	1.19646	BCL2, IL23A
Alcoholism	1.19646	HIST1H3B, HIST2H4B
mTOR signaling pathway	1.16873	HIF1A
Herpes simplex infection	1.14479	C1QBP, GTF2IRD1
Colorectal cancer	1.14163	BCL2
Renal cell carcinoma	1.09045	HIF1A

PPAR signaling pathway	1.06625	ACAA1
Inflammatory bowel disease (IBD)	1.06625	IL23A
Epstein-Barr virus infection	1.0367	BCL2, POLR2J
RNA degradation	1.00939	PABPC1L
Gastric acid secretion	0.987977	KCNK10
Pertussis	0.977536	IL23A
Antigen processing and presentation	0.977536	KIR3DL1
Peroxisome	0.927797	ACAA1
Apoptosis	0.881805	BCL2
Small cell lung cancer	0.881805	BCL2
GABAergic synapse	0.855826	GABARAPL1
Prostate cancer	0.847418	BCL2
Glycerophospholipid metabolism	0.839131	PNPLA6
Morphine addiction	0.83096	ADRBK2
NF-kappa B signaling pathway	0.83096	Bcl2
Rheumatoid arthritis	0.83096	IL23A
Carbon metabolism	0.776864	PCCB
Melanogenesis	0.762334	POMC
MAPK signaling pathway	0.743159	MAP3K8, RASGRP1
Toll-like receptor signaling pathway	0.720971	MAP3K8
Cytokine-cytokine receptor interaction	0.709588	IL15RA, IL23A
TNF signaling pathway	0.701456	MAP3K8
Cholinergic synapse	0.688846	BCL2
Glutamatergic synapse	0.670505	ADRBK2
Neurotrophin signaling pathway	0.641379	BCL2
Toxoplasmosis	0.641379	BCL2
Lysosome	0.630202	AP4B1
MicroRNAs in cancer	0.615074	BCL2, MIR34B
Insulin signaling pathway	0.535764	SLC2A4
Hepatitis B	0.509766	BCL2
Adrenergic signaling in cardiomyocytes	0.501455	BCL2
Pathways in cancer	0.499403	BCL2, HIF1A
Protein processing in endoplasmic reticulum	0.433732	BCL2
Transcriptional misregulation in cancer	0.394724	HIST1H3B
Huntington's disease	0.388639	POLR2J
Chemokine signaling pathway	0.368195	ADRBK2
Endocytosis	0.328479	ADRBK2
Focal adhesion	0.323569	BCL2
Viral carcinogenesis	0.321145	HIST2H4B
Regulation of actin cytoskeleton	0.30475	FGD1
Proteoglycans in cancer	0.282953	HIF1A
Ras signaling pathway	0.276762	RASGRP1
HTLV-I infection	0.213123	IL15RA
Neuroactive ligand-receptor interaction	0.191498	P2RY8
Metabolic pathways	0.135322	ACAA1, GCDH, NME3, PCCB, POLR2J
PI3K-Akt signaling pathway	0.118991	Bcl2
