# Supplementary material for: C1QBP Mediates Breast Cancer Cell Proliferation and Growth via Multiple Potential Signalling Pathways
Source: Int J Mol Sci. 2023 Jan 10;24(2):1343. doi: 10.3390/ijms24021343 (PMC9864289; doi:10.3390/ijms24021343)

Supplementary Fig 1: Validation of up-regulated and down-regulated genes obtained from the Gene ST 2.0 microarray data. The relative gene expression was obtained from real-time PCR and values are presented as mean of triplicates ± SEM. This was compared to the fold change value obtained from the gene microarray. Experiments were done in triplicates and repeated three times.

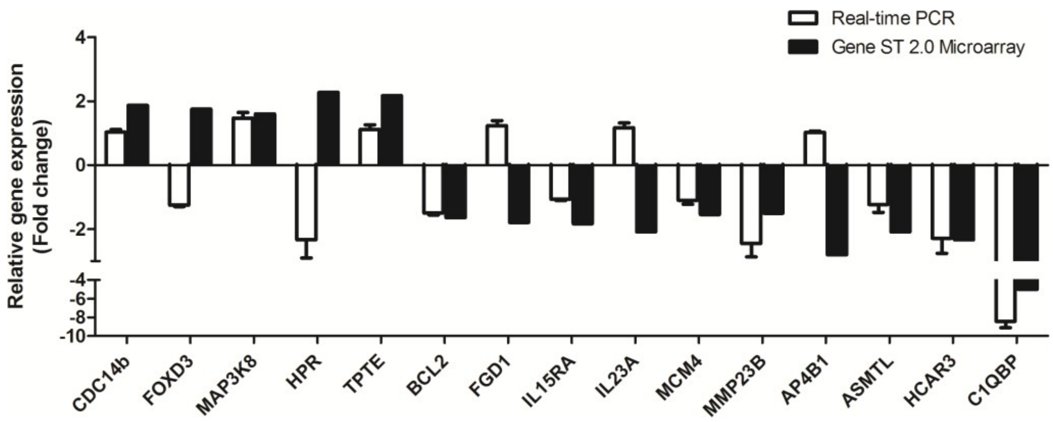

Supplement: Supplementary file 1 [file ijms-24-01343-s001.zip › Supplementary Figure S1.pdf]
